# The Sweet Potato K^+^ Transporter IbHAK11 Regulates K^+^ Deficiency and High Salinity Stress Tolerance by Maintaining Positive Ion Homeostasis

**DOI:** 10.3390/plants12132422

**Published:** 2023-06-23

**Authors:** Hong Zhu, Jiayu Guo, Tao Ma, Shuyan Liu, Yuanyuan Zhou, Xue Yang, Qiyan Li, Kaiyue Yu, Tongshuai Wang, Sixiang He, Chunmei Zhao, Jingshan Wang, Jiongming Sui

**Affiliations:** 1 College of Agronomy, Qingdao Agricultural University, Qingdao 266109, China; zhuhong@qau.edu.cn (H.Z.); 17852845051@163.com (J.G.); mataoqau@163.com (T.M.); rr50053180@163.com (S.L.); yangxue12072022@163.com (X.Y.); lqy183292@163.com (Q.L.); kyyuyuyu@163.com (K.Y.); tongshuai12022@163.com (T.W.); sixiang1106@163.com (S.H.); meiwei2002@163.com (C.Z.); jswang319@163.com (J.W.); 2 Academy of Dongying Efficient Agricultural Technology and Industry on Saline and Alkaline Land in Collaboration with Qingdao Agricultural University, Dongying 257091, China; 3 Crop research Institute, Shandong Academy of Agricultural Sciences/Scientific Observing and Experimental Station of Tuber and Root Crops in Huang-Huai-Hai Region, Ministry of Agriculture and Rural Affairs, Jinan 250100, China; zhou_yy_2020@163.com

**Keywords:** sweet potato, K^+^ transporter, IbHAK11, K^+^ deficiency tolerance, high salinity tolerance, K^+^/Na^+^ homeostasis

## Abstract

The K^+^ transporter KT/HAK/KUP (K^+^ transporter/high-affinity K^+^/K^+^ uptake) family has a critical effect on K^+^ uptake and translocation in plants under different environmental conditions. However, the functional analysis of KT/HAK/KUP members in sweet potatoes is still limited. The present work reported the physiological activity of a new gene, *IbHAK11*, in the KT/HAK/KUP family in sweet potatoes. *IbHAK11* expression increased significantly in the low K^+^-tolerant line compared with the low K^+^-sensitive line following treatment with low K^+^ concentrations. *IbHAK11* upregulation promoted root growth in *Arabidopsis* under low K^+^ conditions. Under high saline stress, transgenic lines had superior growth and photosynthetic characteristics compared with the wild-type (WT). As for *IbHAK11*-overexpressing plants, activation of both the non-enzymatic and enzymatic reactive oxygen species (ROS) scavenging systems was observed. Therefore, *IbHAK11*-overexpressing plants had lower malondialdehyde (MDA) and ROS levels (including H_2_O_2_ and O^2−^) compared with WT under salt-induced stress. We also found that under both low K^+^ and high salinity conditions, overexpression of *IbHAK11* enhanced K^+^ translocation from the root to the shoot and decreased Na^+^ absorption in *Arabidopsis*. Consequently, IbHAK11 positively regulated K^+^ deficiency and high salinity stresses by regulating K^+^ translocation and Na^+^ uptake, thus maintaining K^+^/Na^+^ homeostasis in plants.

## 1. Introduction

Plant growth depends on numerous environmental factors. Abiotic stresses seriously limit plant growth, productivity and quality [1,2]. Soil salinization induced by NaCl is one of the most important in the world [3,4]. As sessile organisms, plants possess numerous mechanisms–such as osmotic balance regulation, ionic homeostasis adjustment, and reactive oxygen species (ROS) scavenging system–to cope with environmental stress during their life cycles [5,6,7]. Apart from osmotic stress, ionic toxicity caused by Na^+^ accumulation is another main adverse effect that occurs under high salinity stress [5]. Adjusting the ionic balance, especially the Na^+^/K^+^ ratio, is one of the most effective means of tolerating salt-induced stress in plants [8,9]. Na^+^ and K^+^ are among the most abundant inorganic cations in the cell cytoplasm and are involved in many basic physiological and metabolic processes in plant development, including stomatal movement, cell elongation, enzyme activation, photosynthesis, osmoregulation and environmental stress adaptation [10,11,12,13]. In plants, K^+^ is an essential nutrient that accounts for approximately 2–10% of the dry weight and its deficiency detrimentally affects plant quality, yield and stress resistance [14,15,16,17,18]. K^+^ modulation in plants is determined by uptake and translocation rather than by metabolization [19]. In contrast to the high K^+^ concentration (100–150 mM) in plant cells, the K^+^ concentration on the soil root surface is low [20]. Consequently, K^+^ deficiency represents the frequently seen abiotic stress; additionally, it can also decrease resistance to other environmental stimuli in plants [19,21]. Plants have developed certain mechanisms of K^+^ acquisition and distribution over their long-term evolution, including K^+^ channels and K^+^ transporters (low- and high-affinity K^+^ uptake systems, respectively) to solve this problem [22,23].

The K^+^ transporters can be classified into four families: KT/HAK/KUP (K^+^ transporter/high-affinity K^+^/K^+^ uptake), KEA (K^+^ efflux anti-porter), CHX (cation/hydrogen exchanger) and Trk/HKT (of which KT/HAK/KUP has the most members in plants) [24,25,26]. Members of the KT/HAK/KUP family exist widely in bacteria, fungi and plants [16,21]. The KT/HAK/KUP genes AtKUP1 in Arabidopsis and HvHAK1 in barley were first isolated in plants based on the conserved sequence of homologs in fungi and bacteria [27,28]. With the developments in genome sequencing techniques, different numbers of KT/HAK/KUP family members located on the inner membranes have been identified in different plants [29], including 13 members in *Arabidopsis*, 27 members in rice and 29 members in soybean [30,31,32]. Members of the KT/HAK/KUP family can be classified into four main clades—named clade I–clade IV—based on their evolutionary relationship [26]. Previous studies showed that many KT/HAK/KUP members in clade I are associated with high-affinity K^+^ uptake, clade II members participate in plant development processes and members in clade III play a role in Na^+^/K^+^ ratio regulation; however, knowledge of the function of clade IV members is limited [26,33,34,35]. In recent years, multiple KT/HAK/KUP family members have been examined and shown to regulate stress resistance in diverse plants, leading to more attention being paid to them. In *Arabidopsis thaliana*, AtHAK5 has a crucial role in high salinity stress tolerance [36]. AtKUP7 acts as a positive regulator under K^+^-limited conditions by mediating K^+^ acquisition and translocation [19]. In addition, AtKUP2, AtKUP6 and AtKUP8 are involved in osmotic stress responses [37]. AtKUP9 maintains root meristem activity under low K^+^ stress by regulating K^+^ and auxin homeostasis [38]. AtKUP12 regulates the Na^+^/K^+^ ratio to enhance salt tolerance [39]. In rice, the clade I members OsHAK1, OsHAK5 and OsHAK21 can enhance Na^+^/K^+^ homeostasis and regulate salt-induced stress tolerance [13,40,41]. OsHAK16 is responsible for K^+^ uptake by the roots as well as for translocation to regulate salt-induced stress tolerance [29]. The KT/HAK/KUP family has also been identified in several other species. For example, SiHAK1 enhances K^+^ uptake in foxtail millet under salt-induced stress, HvHAK1 confers drought resistance by enhancing H^+^ homeostasis in barley and ZmHAK1 and ZmHAK5 function in K^+^ uptake under K^+^-deficient conditions [17,21,42]. Additionally, several reports have shown that the KT/HAK/KUP genes are strongly triggered under abiotic stresses in different plants like cassava, cotton and peach [43,44,45].

Sweet potato (*Ipomoea batatas* (L.) Lam) is a critical feed, food, energy source and industrial crop. It is the sixth most common food crop and has a crucial role in ensuring energy and food security around the world. Since it is usually grown on marginal land, abiotic stresses seriously limit its production [46]. Several genes are associated with tolerance to abiotic stresses [46,47]. A recent study identified 22 KT/HAK/KUP family members in a hexaploidy-cultivated sweet potato and analyzed the expression profiles of these genes [48]. However, the functional analysis of the KT/HAK/KUP gene family members in this crop is still limited.

This work identified a new KT/HAK/KUP gene, *IbHAK11*, in Shangshu19, a variety of sweet potatoes. Its expression in both the root and shoot was significantly different in Shangshu19 (low K^+^ tolerant variety) compared with Yuzi7 (low K^+^ sensitive variety). IbHAK11 confers salt and low K^+^ stress tolerance by regulating K^+^ translocation in transgenic plants. This study provides information for further functional and mechanism analyses of abiotic stress tolerance by the KT/HAK/KUP family in sweet potatoes and other plants.

## 2. Results

### 2.1. Characterization of IbHAK11

**Figure 1:**
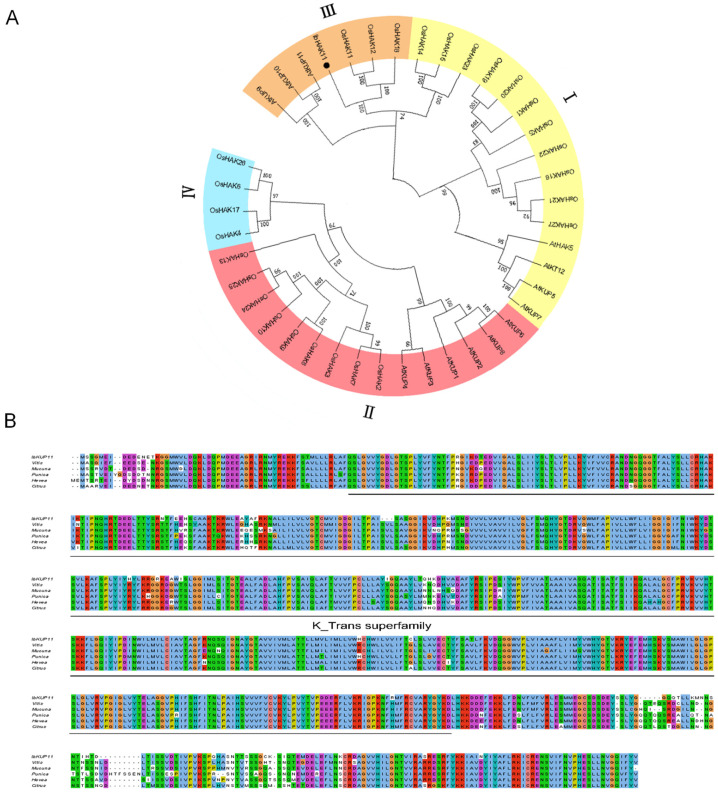

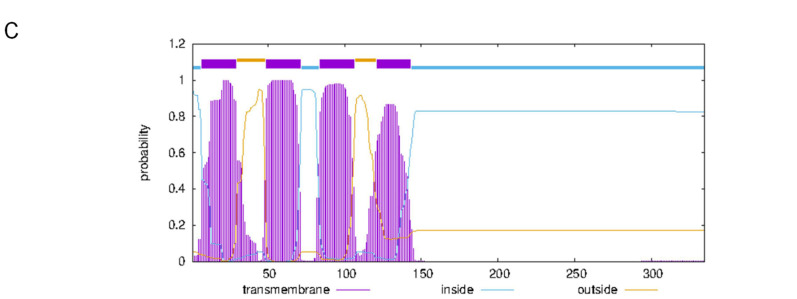
Phylogenetic and amino acid sequence analysis of IbHAK11. (**A**) Phylogenetic analysis of IbHAK11, 13 KT/HAK/KUP members from Arabidopsis and 27 KT/HAK/KUP members from rice. (**B**) Alignment of IbHAK11 amino acid sequence against NCBI-derived homologs. (**C**) Transmembrane domain prediction in IbHAK11.

The 1008 bp full-length ORF of *IbHAK11* was isolated from Shangshu19, a low K^+^ tolerant sweet potato (unpublished data). The IbHAK11 protein consists of 335 amino acids (molecular weight: 38.154 kDa; isoelectric point (pI): 8.29). Phylogenetic analysis was conducted using 41 KT/HAK/KUP members, including 13 from *Arabidopsis*, 27 from rice and 1 from sweet potatoes. The results showed that the 41 KT/HAK/KUP members could be divided into four subgroups, with the 13 KT/HAK/KUP members from *Arabidopsis* being distributed across three of the subgroups (Figure 1A). IbHAK11 shared a higher similarity with AtHAK11 than with other family members and was classified into subgroup III with AtHAK11 and OsHAK11 (Figure 1A). Multiple alignments of IbHAK11 and its homologs from other species suggested that IbHAK11 was homologous to KT/HAK/KUP members from *Vitis riparia*, *Mucuna pruriens*, *Punica granatum*, *Hevea brasiliensis* and *Citrus sinensis*, belonging to subgroup III of the KT/HAK/KUP family (Figure 1B). The prediction of protein structure showed that four predicted transmembrane domains were present in IbHAK11, which indicates that it is a membrane-localizing protein (Figure 1C).

### 2.2. Expression Profiles of IbHAK11

**Figure 2:**
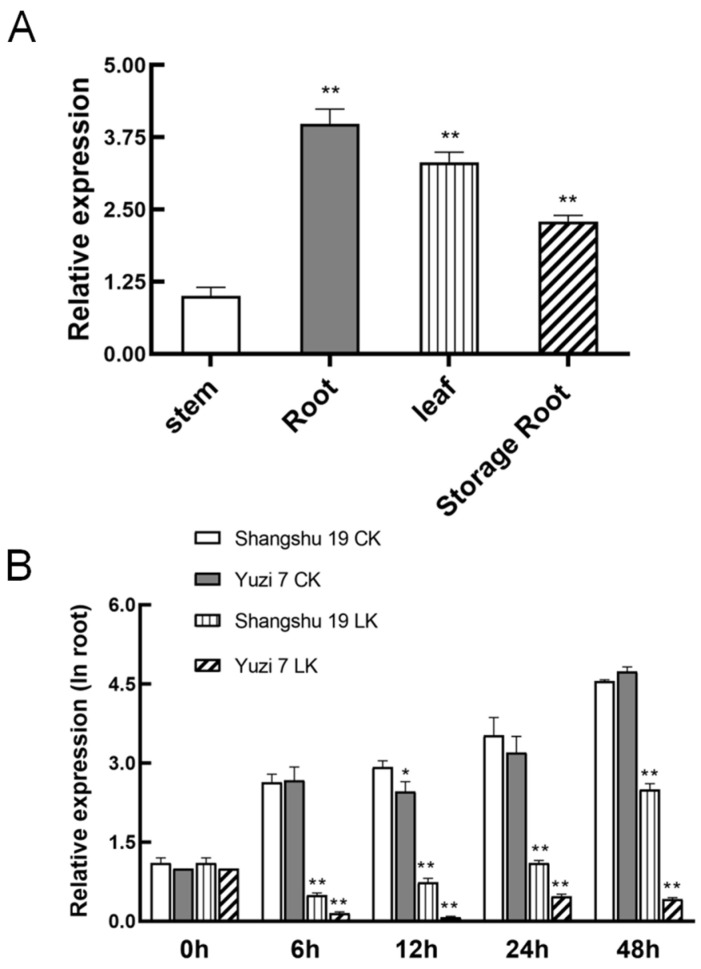

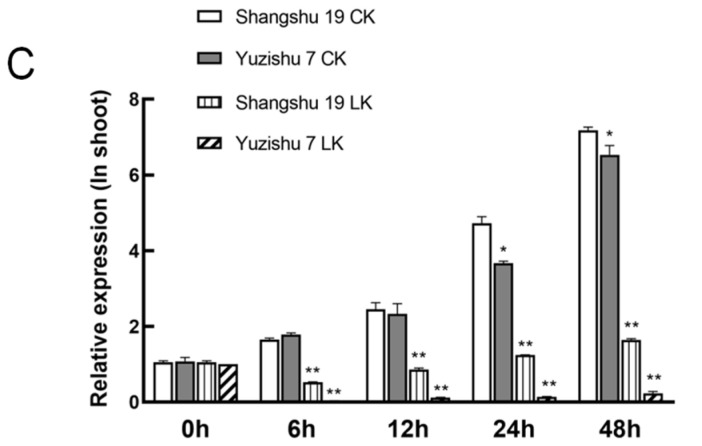
Analysis of *IbHAK11* expression. (**A**) Expression profiles of *IbHAK11* in different tissues. Expression patterns of *IbHAK11* in (**B**) roots and (**C**) shoots following treatment with low or normal K^+^ concentrations. Results are presented as mean ± SE (*n* = 3). * *p* < 0.05 and ** *p* < 0.01 represent statistical significance.

To analyze the specific expression patterns of *IbHAK11*, four different Shangshu19 tissues were used for qRT-PCR assay. The results showed that IbHAK11 was expressed in all the tested tissues but at different levels (Figure 2A). The highest expression level of this gene was detected in the roots, which is one of the most important tissues responsible for K^+^ uptake and translocation (Figure 2A). For low K^+^ stress response analysis, *IbHAK11* levels in the roots and shoots of the low K^+^-tolerant Shangshu19 line and the low K^+^-sensitive Yuzi7 line were determined following normal K^+^ and low K^+^ treatments over different lengths of time. Upon treatment with normal K^+^ concentrations, *IbHAK11* levels in both roots and shoots of Shangshu19 and Yuzi7 stabilized (Figure 2B,C). However, under low K^+^ conditions, *IbHAK11* expression in the roots and shoots of Shangshu19 and Yuzi7 decreased (Figure 2B,C). However, its levels in both roots and shoots increased significantly in Shangshu19 compared with Yuzi7 (Figure 2B,C). Based on these results, *IbHAK11* has a possible critical effect on tolerance to low K^+^-induced stress.

### 2.3. Overexpression of IbHAK11 Confers Low K^+^ Tolerance to Transgenic Plants

**Figure 3:**
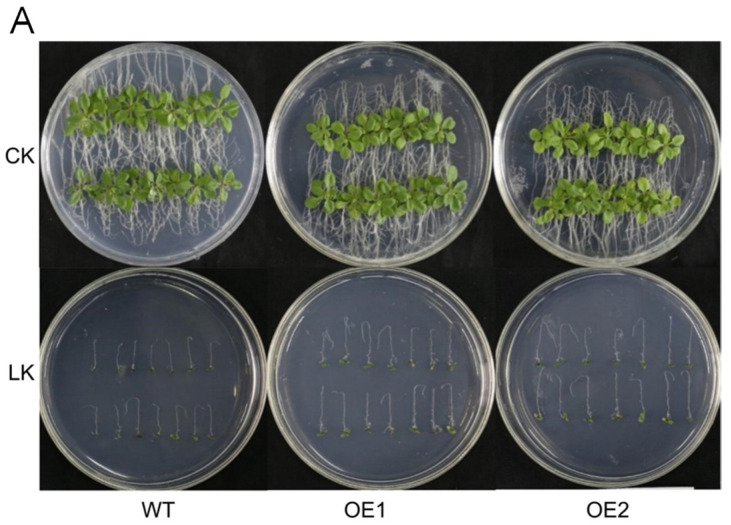

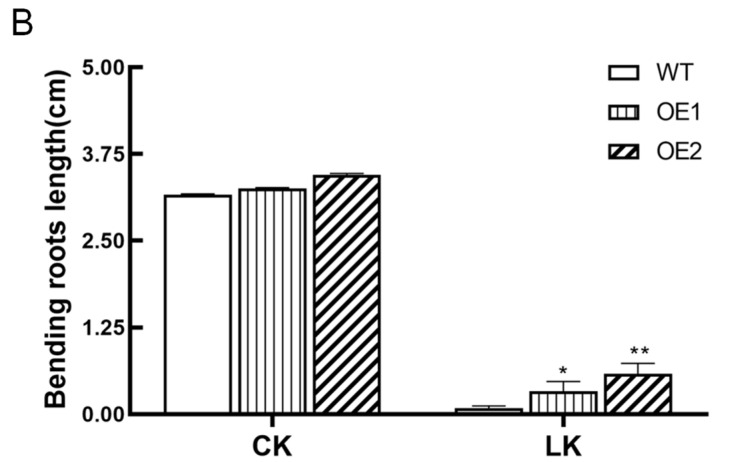
Responses in WT and transgenic seedlings cultivated under low K^+^ and normal conditions for 10 days. (**A**) WT and transgenic plant phenotypes. (**B**) Bending root lengths in WT and transgenic plants. Results are presented as mean ± SE (*n* = 3). * *p* < 0.05 and ** *p* < 0.01 represent statistical significance.

To investigate the physiological function of *IbHAK11*, 15 independent *Arabidopsis* lines overexpressing this gene were obtained and confirmed using PCR (Figure S1). We randomly screened two homozygous transgenic lines (OE1 and OE2) to conduct further low K^+^ tolerance assays. After germination, we transferred WT and *IbHAK11* transgenic lines into a 1/2 MS medium containing K^+^ concentrations of 10 mM (normal conditions) and 50 μM (low K^+^). Due to geotropism, new roots curved downward when the plantlets were cultured upside down. Under normal conditions, WT and transgenic lines showed favorable growth, with no differences in morphology; however, limited root development was observed in all tested plants after treatment with low K^+^ for 10 days (Figure 3A). The length of bending roots was significantly lower in both WT and transgenic plantlets under low K^+^ conditions compared with normal conditions (Figure 3B). Nevertheless, the bending root of the transgenic lines showed better growth compared with WT (Figure 3B). These results indicate that overexpression of *IbHAK11* conferred tolerance to low K^+^ in transgenic *Arabidopsis*.

### 2.4. IbHAK11 Enhances Resistance to High Salinity in Overexpressing Lines

**Figure 4:**
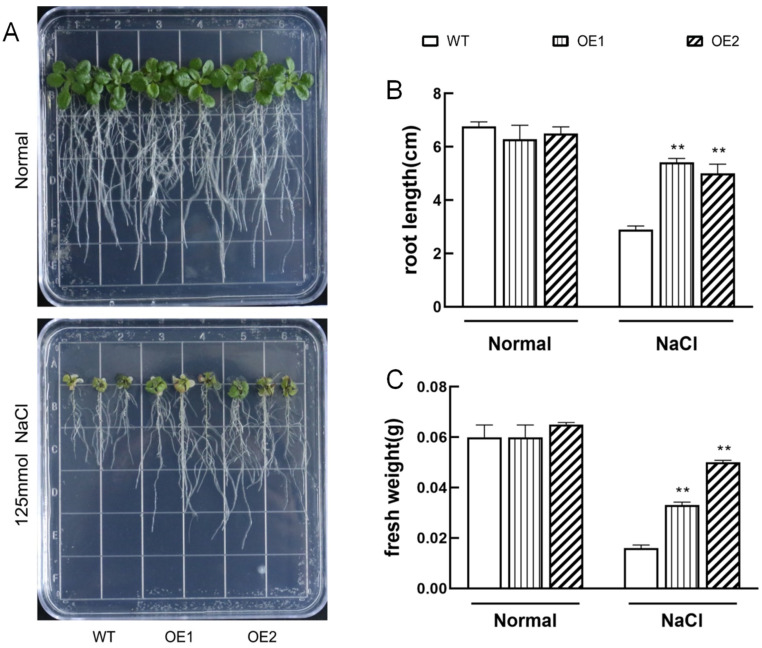
Responses in WT and transgenic seedlings cultivated for 15 days in 1/2 MS medium in the absence of stress or in the presence of 125 mM NaCl. (**A**) Morphologies of WT and transgenic lines. (**B**) Primary root lengths. (**C**) Fresh weight. Results are presented as mean ± SE (*n* = 3). ** *p* < 0.01 represents statistical significance.

**Figure 5:**
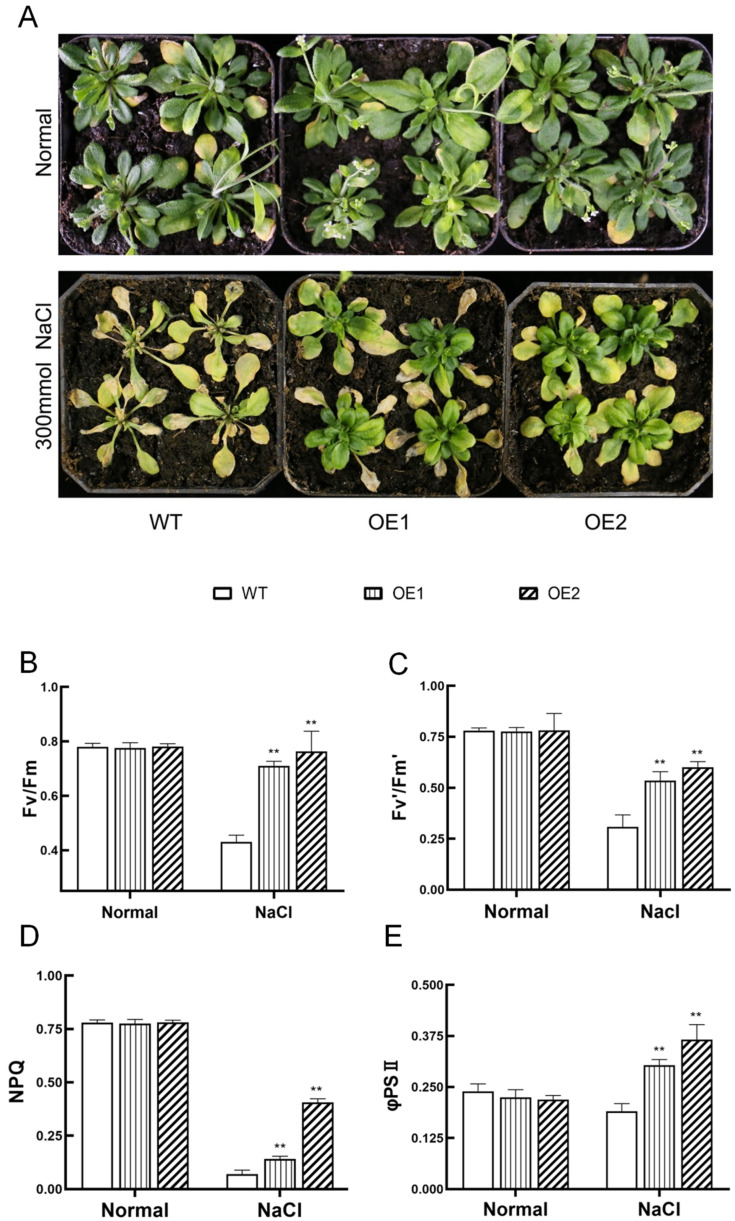
Responses in WT and transgenic lines cultured in pots under high-salinity and normal conditions. (**A**) Morphologies of plants exposed to different conditions. Plant photosynthetic parameters such as (**B**) Fv/Fm, (**C**) Fv’/Fm’, (**D**) NPQ and (**E**) φPSII. Results are presented as mean ± SE (*n* = 3). ** *p* < 0.01 represent statistical significance.

Since overexpression of *IbHAK11* conferred tolerance to low K^+^, we also investigated tolerance to high salinity stress in transgenic lines. Obvious morphological differences were not observed between WT and *IbHAK11* transgenic lines in a normal 1/2 MS medium (Figure 4A). Transgenic lines had higher fresh weight and root lengths compared with WT grown on 1/2 MS medium containing 125 mM NaCl, which matched the results of the morphological observations (Figure 4A–C). Furthermore, tolerance to high salinity in *IbHAK11* transgenic lines was studied using plantlets grown in pots containing soil mixtures. Under normal irrigation conditions, both WT and transgenic plants grew exuberantly and rapidly (Figure 5A). Chlorophyll fluorescence results also revealed the absence of differences in maximal photosystem II (PSII) photochemical efficiency in the dark (Fv/Fm), PSII photochemical efficiency in the light (Fv’/Fm’), real PSII efficiency (φPSII) and non-photochemical quenching of PSII (NPQ) (Figure 5B–E). Upon exposure to high salinity stress, parameters—including chlorophyll fluorescence—all significantly increased in the overexpressing plants compared with the WT (Figure 5B–E). Therefore, *IbHAK11* upregulation enhanced high salinity stress tolerance in transgenic *Arabidopsis*.

### 2.5. IbHAK11 Maintains ROS Homeostasis under Salt-Induced Stress

**Figure 6:**
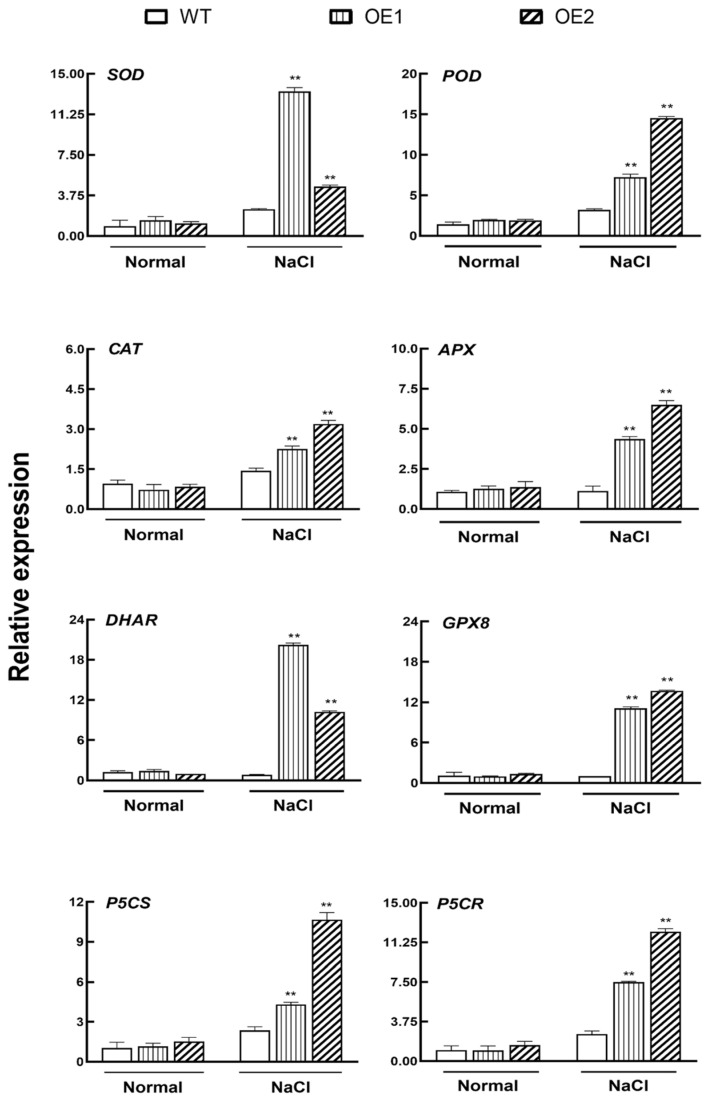
Expression of ROS scavenging-related gene transcripts in WT and transgenic lines. Results are presented as mean ± SE (*n* = 3). ** *p* < 0.01 represents statistical significance.

Under optimal growth conditions, cellular ROS content is low; however, the level increases following exposure to abiotic stress [49]. Accumulation of excess ROS has deleterious effects on membranes and biological molecules, resulting in cellular damage and death through lipid peroxidation [50]. To determine ROS homeostasis levels, expression profiles of several ROS scavenging-related genes, including non-enzymatic and enzymatic genes were examined using qRT-PCR. As a result, *AtSOD*, *AtPOD*, *AtCAT*, *AtAPX*, *AtDHAR* and *AtGPX8*, which encode ROS scavenging enzymes, and *AtP5CR* and *AtP5CS*, which are involved in proline biosynthesis, were significantly upregulated in transgenic plantlets compared with WT following exposure to high salinity stress (Figure 6). However, these genes were present at similar levels in the WT and the two overexpressing plants following normal irrigation conditions (Figure 6). These results show that overexpression of *IbHAK11* activates stress-responsive genes under high salinity stress.

**Figure 7:**
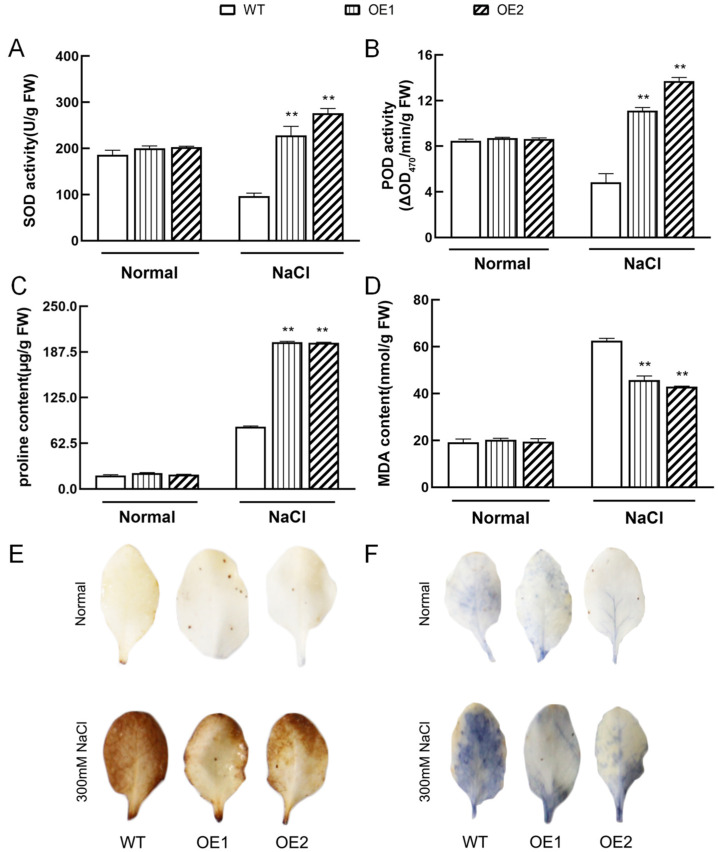
Expression of ROS scavenging-related gene transcripts in WT and transgenic lines. (**A**) SOD activity. (**B**) POD activity. (**C**) Proline levels. (**D**) MDA levels. Results are presented as mean± SD (*n* = 3). ** *p* < 0.01 represents statistical significance in WT plants compared with transgenic plants. (**E**) DAB and (**F**) NBT staining of leaf samples following exposure to normal and high salinity conditions.

Furthermore, we analyzed the activities of ROS scavenging enzymes, including SOD and POD, and proline levels. Compared with the WT, higher SOD and POD activities were observed in *IbHAK11*-overexpressing lines (Figure 7A–C). Since malondialdehyde (MDA) is a parameter of membrane damage caused by ROS mediating lipid peroxidation, its content was also measured in this study. The results showed that lower MDA content was detected in transgenic plants under salt treatment, which suggests better stability and integrity of cell membranes (Figure 7D). Additionally, H_2_O_2_ and O^2−^ accumulation in leaf samples was examined visually using 3,3′-diaminobenzidine (DAB) and Nitro-blue tetrazolium chloride (NBT) histochemical staining, respectively. H_2_O_2_ and O^2−^ accumulation was lower in both WT and transgenic plants following exposure to normal irrigation conditions (Figure 7E,F). Based on the DAB and NBT staining results, under salt stress, IbHAK11 upregulation reduced the accumulation of H_2_O_2_ and O^2−^ (Figure 7E,F). These results collectively demonstrate that IbHAK11 positively regulates ROS scavenging to maintain ROS homeostasis and enhance resistance to high salinity.

### 2.6. IbHAK11 Regulates K^+^ Uptake and Translocation under Low K^+^ or High Salinity Conditions

**Figure 8:**
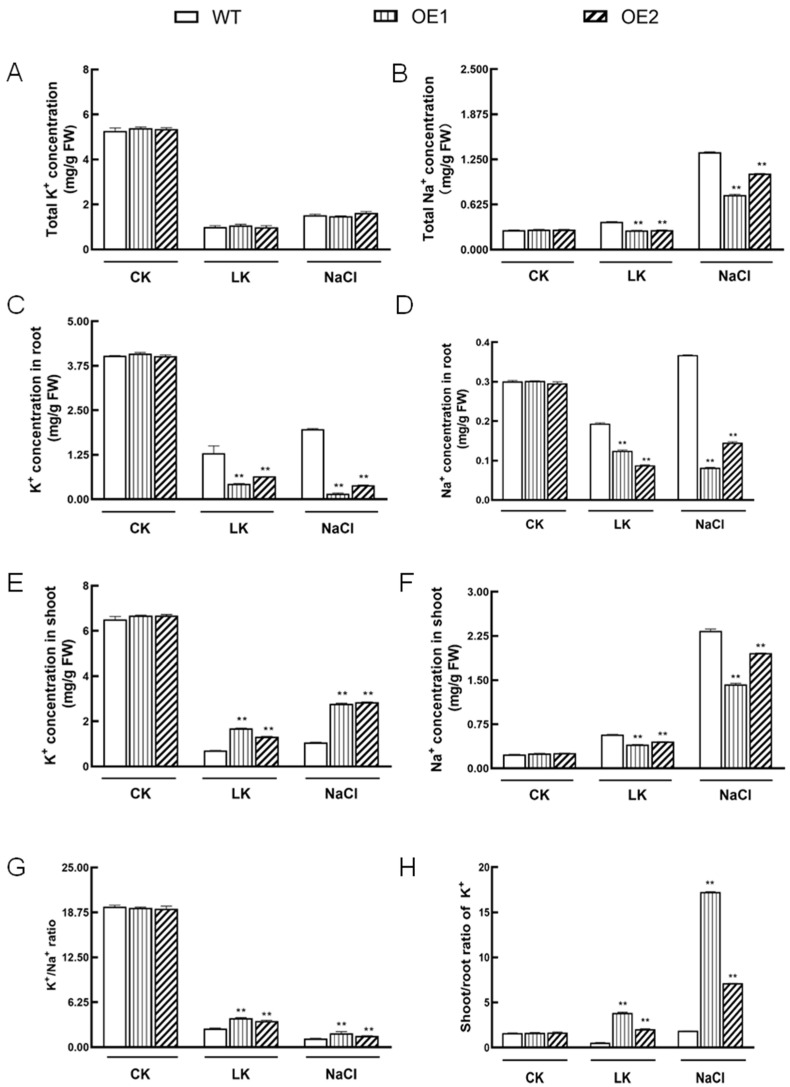
K^+^ and Na^+^ concentrations in WT and transgenic lines following exposure to normal, low K^+^ and high salinity conditions. K^+^ (**A**) and Na^+^ (**B**) contents in entire plantlets. K^+^ (**C**) and Na^+^ (**D**) contents in roots. K^+^ (**E**) and Na^+^ (**F**) contents in shoots. K^+^/Na^+^ ratio (**G**) in all plants. Shoot/root K^+^ ratio (**H**). The results are presented as mean ± SD (*n* = 3). ** *p* < 0.01 represents statistical significance in WT plants compared with transgenic plants.

Previous studies indicated that KT/HAK/KUP members belonging to different clades might play varying roles in stress responses [51]. To study the mechanism of action of IbHAK11 under different stress treatments, K^+^ and Na^+^ contents in WT and transgenic lines were measured. Following treatment with low K^+^, the K^+^ concentration in the whole plant decreased in WT and transgenic plants, which revealed the absence of significant differences among the tested plantlets (Figure 8A). K^+^ levels in all plants treated with NaCl increased compared with plants treated with low K^+^; however, they were still lower than the levels in plants grown in normal 1/2 MS medium (Figure 8A). Analysis of Na^+^ showed that Na^+^ content decreased in transgenic plants relative to WT plants following exposure to low K^+^ (Figure 8B). Under high salinity stress, accumulation of Na^+^ increased in WT plants compared with plants grown in normal 1/2 MS medium; however, this was still lower than in transgenic *Arabidopsis* (Figure 8B). This suggests that IbHAK11 can regulate Na^+^ uptake, to some degree. K^+^ and Na^+^ concentrations in the shoots and roots of WT and transgenic plants were measured. In roots, K^+^ levels in all tested plants decreased following exposure to low K^+^ and high salinity conditions; this decrease in both *IbHAK11*-overexpressing lines and WT following stress treatment was marked. Na^+^ accumulation also significantly decreased in transgenic plants after exposure to low K^+^ or high salinity (Figure 8C,D). In shoots, K^+^ accumulation increased significantly in *IbHAK11*-overexpressing plants relative to WT plants following exposure to low K^+^ conditions; however, Na^+^ levels markedly decreased in transgenic plants (Figure 8E,F). K^+^/Na^+^ values showed that overexpression of *IbHAK11* enhanced the K^+^/Na^+^ ratio in transgenic plants compared with WT plants under low K^+^ or high salinity (Figure 8G). Furthermore, the shoot/root ratio of K^+^ was quantified under different growth conditions. The results showed that the shoot/root ratio of K^+^ increased in shoots but decreased in roots under stress conditions due to the overexpression of *IbHAK11* (Figure 8H). These results show that IbHAK11 regulates resistance to low K^+^ and high salinity by mediating Na^+^ uptake and K^+^ translocation.

## 3. Discussion

### 3.1. IbHAK11 Enhanced Resistance to K^+^ Deficiency via Na^+^ Uptake and K^+^ Translocation

As an essential plant nutrient, K^+^ plays key roles in many biological processes, including cell growth, enzyme activity, transcription, post-translational modification and stress responses [52]. Since K^+^ cannot be metabolized by plant cells, it is mostly regulated via environmental acquisition [19]. The K^+^ content in plant cells is significantly higher relative to the content in the soil, thus K^+^ deficiency represents a frequently seen abiotic stress encountered by plants [21]. To deal with this problem, plants have evolved a series of mechanisms to increase K^+^ utilization efficiency [26,53]. Many previous studies have suggested that K^+^ transporters play major roles in maintaining cation homeostasis by regulating K^+^ uptake and translocation [54]. AKT1, which is an inward-rectifying shaker K^+^ channel, and HAK5, which is a high-affinity K^+^ transporter, represent two main components that contribute to K^+^ acquisition, and the expression of *AKT1* and *HAK5* can be induced by K^+^ deficient treatment [55,56]. Several members of the KT/HAK/KUP family act as high-affinity K^+^ transporters, for example, HvHAK1 in barley and OsHAK1 in rice [27,57]. However, not all KT/HAK/KUP members are candidates for high-affinity K^+^ transporters, indicating their different functions under different conditions [19]. In this study, we reported a newly identified K^+^ transporter in sweet potatoes called IbHAK11, which shared the highest homology with AtHAK11 (Figure 1A,B). Its transcription level significantly increased in low K^+^-tolerant plants relative to low K^+^-sensitive plants (Figure 2B). Based on analyses of expression profiles in previous studies, we predicted that IbHAK11 also functions in tolerance to K^+^ deficiency [40,56]. Further studies demonstrated that overexpression of *IbHAK11* enhanced low K^+^ tolerance in transgenic Arabidopsis (Figure 3).

It has been reported that several members function in K^+^ acquisition at different K^+^ levels. Previous studies showed that in *Arabidopsis*, AtHAK5 and AtKUP7 exhibited different affinities for K^+^ absorption [19,58]. Additionally, AtKUP1 functioned at both high and low K^+^ levels [59]. *OsHAK1* levels in rice roots were triggered under K^+^ deficiency conditions, which assumed about 50–55% of K^+^ uptake under low K^+^ (0.05–0.1 mM) conditions [41]. In the present study, K^+^ concentration in the whole plant was not significantly different in WT compared with transgenic lines under K^+^ deficiency conditions, which suggests that IbHAK11 had little effect on K^+^ absorption under low K^+^ stress (Figure 8A). After absorption, K^+^ can be translocated from the root to the shoot. In *Arabidopsis*, AtKUP7 may participate in the long-distance transport of K^+^ under K^+^-deficient conditions [19]. OsHAK5 may also be involved in K^+^ translocation from the root to the shoot in rice [40]. In this study, the K^+^ concentration was significantly higher in the shoots than in the roots of transgenic lines compared with WT lines (Figure 8C,E). At the same time, the Na^+^ concentration was lower in *IbHAK11*-overexpressing lines under low K^+^ stress (Figure 8B,D,F). These results indicate that IbHAK11 enhanced resistance to K^+^ deficiency by regulating Na^+^ uptake and K^+^ translocation rather than K^+^ absorption to maintain ion homeostasis.

### 3.2. IbHAK11 Enhanced Tolerance to High Salinity by Regulating ROS Scavenging and Ion Homeostasis

High salinity accounts for the main abiotic stress that threatens crop productivity worldwide. Following exposure to salt-induced stress, excess Na^+^ accumulates, causing cellular toxicity [5]. Previous studies showed that K^+^ leakage and generation of ROS resulted in cell death under high salinity stress [60]. Maintaining the K^+^/Na^+^ ratio is considered an important strategy for resisting high salinity stress in plants [61]. Therefore, several members associated with salt-induced Na^+^ and K^+^ transport regulated tolerance to salt-induced stress [9,29,38]. The present study showed that under salt-induced stress, overexpressing plants had superior growth compared with WT plants (Figure 4A–C and Figure 5A). The results of chlorophyll fluorescence analysis also verified the morphological features (Figure 5B–E). These results suggest that overexpression of IbHAK11 positively regulates tolerance to salt-induced stress in *Arabidopsis*. In rice, OsHAK16 positively regulated the K^+^/Na^+^ ratio and the shoot/root ratio of K^+^ to enhance tolerance to high salinity [29]. SlHAK20 conferred salt tolerance by mediating the K^+^/Na^+^ ratio in tomatoes [9]. Several KT/HAK/KUP members are differentially expressed in sweet potatoes under salt-induced stress; however, the functional analysis of these genes is limited [48]. Analysis of Na^+^ and K^+^ concentrations showed that overexpression of *IbHAK11* enhanced K^+^ translocation from the root to the shoot, and the K^+^/Na^+^ ratio under high salinity (Figure 8A,G,H). It is worth noting that IbHAK11 also regulated Na^+^ absorption under salt-induced stress (Figure 8B,D,F). These results collectively suggest that the mechanism of action of IbHAK11 in K^+^ regulation is similar under low K^+^ and high Na^+^ conditions, and the K^+^/Na^+^ ratio might be the trigger.

The accumulation of ROS is a common consequence in plants exposed to high salinity [5]. Excessive ROS has deleterious impacts on biological molecules, resulting in cellular damage and death [62]. Increasing evidence suggests that the K^+^/Na^+^ ratio, rather than the absolute quantity of K^+^ and Na^+^, influences tolerance to salinity-induced stress [63]. It has been demonstrated that the NaCl-mediated K^+^ efflux in leaf mesophylls is mainly mediated by NSCC (non-selective cation) as well as KOR (K^+^ outward rectifying) channels elevated with ROS [64]. Earlier studies also suggested a positive correlation between ROS and K^+^/Na^+^ balance [65,66]. In this study, the levels of several non-enzymatic and enzymatic ROS scavenging-system-related genes were markedly upregulated in transgenic lines compared with the WT (Figure 6). Accordingly, SOD and POD activities and proline content in transgenic lines markedly increased relative to WT lines upon exposure to salt-induced stress (Figure 7A–C). As a result of enhanced ROS scavenging, ROS accumulation in transgenic leaves, including H_2_O_2_ and O^2−^, was less than in WT, as visualized by DAB and NBT staining, respectively (Figure 7E,F). Therefore, *IbHAK11* upregulation promoted ROS scavenging following exposure to high salinity stress to enhance abiotic resistance.

## 4. Materials and Methods

### 4.1. Plant Materials

The low K^+^-tolerant Shangshu19 variety and the low K^+^-sensitive Yuzi7 variety were used for the analysis of *IbHAK11* expression (unpublished data). In addition, this work used *Arabidopsis thaliana* (Columbia-0) to genetically transform *IbHAK11*. Growth performances of both sweet potatoes and *Arabidopsis* were similar to those described by Zhu et al. [67].

### 4.2. IbHAK11 Sequencing

Total RNA was isolated from Shangshu19 using RNAprep Pure Plant Kit (Tiangen Biotech, Beijing, China). The PrimeScript™ II 1st Strand cDNA Synthesis Kit (TaKaRa, Beijing, China) was used to synthesize first-strand cDNA. Experiments were conducted in line with specific instructions. Coding sequences (CDs) of *IbHAK11* were amplified using IbHAK11-F/R (Supplementary Table S1). The website ExPASy (https://web.expasy.org/protparam/) (accessed on 1 July 2020) was used to predict IbHAK11 based on its isoelectric point and molecular weight. Phylogenic analysis of IbHAK11, AtKT/HAK/KUPs from *Arabidopsis* and OsKT/HAK/KUPs from rice was performed using the neighbor-joining method implemented in the MEGA 6.0 software. The DNAMAN 8.0 software was used to conduct protein sequence alignment of IbHAK11 and its homologous proteins from other species. The web-based TMHM Server (http://www.cbs.dtu.dk/services/TMHMM-2.0/) (accessed on 1 July 2020) was used to predict IbHAK11’s transmembrane domain.

### 4.3. IbHAK11 Expression Profiling

*IbHAK11* expression was analyzed in different tissues, including leaves, stems, fibrous roots and storage roots, of a 3-month-old field-grown Shangshu19. Additionally, 4-week-old in vitro Shangshu19 and Yuzi7 plantlets were subjected to Hoagland solution treatment containing 0/20 mM K^+^ to determine *IbHAK11* expression in roots and shoots in response to low K^+^ stress. Sampling was conducted at 0/6/12/24/48 h after treatment and samples were analyzed using quantitative real-time polymerase chain reaction (qRT-PCR) and the 2^−ΔΔCT^ method as described by Zhang et al. [46]. The QuantStudio 3 (Applied Biosystems, Foster City, CA, USA) and TB Green Premix Ex Taq^TM^ II Kit (TaKaRa, Beijing, China) were used to conduct qRT-PCR analysis, with IbActin as the endogenous reference. Supplementary Table S1 lists the sequences of the specific primers used.

### 4.4. Production of IbHAK11 Transgenic Plants

After restriction enzyme digestion, the entire open reading frame (ORF) of *IbHAK11* containing the specific primer (IbHAK11-OE-F/R)-amplified restriction enzyme cutting sites was cloned into the expression vector pCAMBIA1300 (Supplementary Table S1). The recombinant vector was transfected into *Agrobacterium tumefaciens* strain GV3101, and *Arabidopsis* was genetically transformed according to Clough et al.’s description [68]. Positive transgenic lines were then selected in a 1/2 Murashige and Skoog (MS) medium containing 50 mg/L hygromycin, and PCR was conducted for identification. Supplementary Table S1 shows all the primers used.

### 4.5. Assay for Low K^+^ and Tolerance to High Salinity

For the low K^+^ tolerance assay, wild-type (WT) and T3 transgenic plant seeds were germinated in 1/2 MS medium after surface sterilization. Seedlings that were 5 days old and had 1 cm-long roots were added to 1/2 MS medium containing 50 μM (low K^+^) or 10 mM (normal conditions) K^+^. The plantlets were cultured upside down for 10 days at 22 °C under 16 h of daylight and new downward-curving roots measured [69].

For the high salinity tolerance assay, after germinating for 7 days in 1/2 MS medium, WT and transgenic plants were added to 1/2 MS medium containing 0/125 mM NaCl. Root length and fresh weight were measured following 15 days of treatment. Additionally, 10-day-old seedlings were cultivated in 1/2 MS medium and then added to a potting soil mixture (vermiculite:rich soil = 1:3, *v*/*v*) and irrigated with 300 mM NaCl solution at 3-day intervals for 2 weeks [70].

### 4.6. Measurement of Photosynthetic Characteristics

The pot-grown plantlets grown under different conditions were used for the determination of photosynthetic parameters. Before taking measurements, the plants were kept in the dark for 40 min. Maximal photosystem II (PSII) photochemical efficiency in the dark (Fv/Fm), PSII photochemical efficiency in the light (Fv’/Fm’), non-photochemical quenching of PSII (NPQ) and real PSII efficiency (φPSII) were measured using IMAG-MAXI (Heinz Walz, Effeltrich, Germany).

### 4.7. Stress-Responsive Gene Levels following Exposure to Salt-Induced Stress

WT and transgenic plants exposed to normal and high salinity treatments were used to analyze stress response-related gene levels using qRT-PCR. RNA was isolated, first-strand cDNA synthesized and qRT-PCR performed as described above, with the *Arabidopsis* actin gene being adopted for normalization of expression levels. Supplementary Table S1 shows the specific primers used.

### 4.8. Determination of ROS Levels and ROS Scavenging System under Salt-Induced Stress

Hydrogen peroxide (H_2_O_2_) and O^2−^ accumulation in transgenic and WT leaves exposed to different conditions were visualized using DAB and NBT staining, respectively [71,72]. Superoxide dismutase (SOD) and peroxidase (POD) activities and MDA and proline levels were determined using the corresponding kits (Suzhou Grace Biotechnology Co., Suzhou, China). All experimental procedures were conducted according to the instructions.

### 4.9. Quantification of Na^+^ and K^+^

Root and shoot samples were gathered to measure K^+^ and Na^+^ concentrations. After washing with double-distilled water, samples were dried for 24 h at 106 °C to obtain dry samples and then dissolved in HNO_3_ and HClO_4_ for 12 h. K^+^ and Na^+^ contents were then determined using the ICO-OES (OPTMA8000DV; PerkinElmer), in line with specific protocols.

### 4.10. Statistical Analysis

The treatments were conducted separately in triplicate. All results are presented as mean ± SE. Student’s *t*-test was performed using SPSS 17.0 software. * *p* < 0.05 and ** *p* < 0.01 represented statistical significance between transgenic plants and WT plants exposed to the corresponding diverse treatments.

## 5. Conclusions

In summary, a new gene that encodes a K^+^ transporter in the KT/HAK/KUP family was isolated and named IbHAK11. Its transcription level in sweet potatoes significantly increased in the low K^+^-tolerant line compared with the low K^+^-sensitive line. In *Arabidopsis*, IbHAK11 enhanced resistance to stress induced by K^+^ deficiency by regulating K^+^ translocation from the root to the shoot and decreasing Na^+^ absorption. Additionally, IbHAK11 enhanced resistance to high salinity stress in transgenic lines, leading to activation of the enzymatic and non-enzymatic ROS scavenging systems. Under salt-induced stress treatment, IbHAK11 showed mechanisms of K^+^ and Na^+^ regulation that were similar to those observed under low K+ conditions. These results indicate that IbHAK11 enhanced abiotic stress tolerance by maintaining K^+^/Na^+^ balance in plants. In conclusion, this study identified a novel KT/HAK/KUP gene for molecular screening of ion stress, including low K^+^ and high salinity, in sweet potatoes and other plants.

## Data Availability

The datasets generated during and/or analyzed during the current study are available from the corresponding author upon reasonable request. The accession number for *IbHAK11* in the sweet potato database (http://ipomoea-genome.org) (accessed on 4 May 2018) is g48527.t1.

## Supplementary Materials

The following supporting information can be downloaded at: https://www.mdpi.com/article/10.3390/plants12132422/s1, Figure S1: PCR identification of *IbHAK11* transgenic plants; Table S1: Primers used in this study.

## References

[1] Gong Z., Xiong L., Shi H., Yang S., Herrera-Estrella L.R., Xu G., Chao D., Li J., Wang P.Y., Qin F. (2020). Plant abiotic stress response and nutrient use efficiency. Sci. China Life Sci..

[2] Kumari P., Rastogi A., Yadav S. (2020). Effects of Heat stress and molecular mitigation approaches in orphan legume, Chickpea. Mol. Biol. Rep..

[3] Kul R. (2022). Integrated application of plant growth promoting rhizobacteria and biochar improves salt tolerance in eggplant seedlings. Turk. J. Agric. For..

[4] Addique M., Kausar A., Iqra I., Akhter N., Mujahid N., Parveen A., Zaman Q., Hussain S. (2022). Amino acids application alleviated salinity stress in spinach (*Spinacia oleracea* L.) by improving oxidative defense, osmolyte accumulation, and nutrient balance. Turk. J. Agric. For..

[5] Zhu J. (2016). Abiotic stress signaling and responses in plants. Cell.

[6] Choudhury F.K., Rivero R.M., Blumwald E., Mittler R. (2017). Reactive oxygen species, abiotic stress and stress combination. Plant J..

[7] Huang X., Mohsin T., Yu M., Sergey S. (2022). Melatonin as a regulator of plant ionic homeostasis: Implications for abiotic stress tolerance. J. Exp. Bot..

[8] Munns R., Tester M. (2008). Mechanisms of salinity tolerance. Annu. Rev. Plant Biol..

[9] Wang Z., Hong Y., Zhu G., Li Y., Niu Q., Yao J., Hua K., Bai J., Zhu Y., Shi H. (2020). Loss of salt tolerance during tomato domestication conferred by variation in a Na^+^/K^+^ transporter. EMBO J..

[10] Clarkson D.T., Hanson J.B. (1980). The mineral nutrition of higher plants. Annu. Rev. Plant Physiol..

[11] Leigh R.A., Jones R.G.W. (1984). A hypothesis relating critical potassium concentrations for growth to the distribution and functions of this ion in the plant cell. New Phytol..

[12] Hedrich R. (2012). Ion channels in plants. Physiol. Rev..

[13] Li W., Xu G., Alli A., Yu L. (2018). Plant HAK/KUP/KT K^+^ transporters: Function and regulation. Semin. Cell Dev. Biol..

[14] Ashley M.K., Grant M., Grabov A. (2006). Plant responses to potassium deficiencies: A role for potassium transport proteins. J. Exp. Bot..

[15] Watanabe T., Broadley M.R., Jansen S., White P.J., Takada J., Satake K., Takamatsu T., Tuah S.J., Osaki M. (2007). Evolutionary control of leaf element composition in plants. New Phytol..

[16] Pettigrew W.T. (2008). Potassium influences on yield and quality production for maize, wheat, soybean and cotton. Physiol. Plant..

[17] Qin Y., Wu W., Wang Y. (2019). *ZmHAK5* and *ZmHAK1* function in K^+^ uptake and distribution in maize under low K^+^ conditions. J. Integr. Plant Biol..

[18] Lotfi R., Abbasi A., Kalaji H.M., Eskandari I., Sedghieh V., Khorsandi H., Sadeghian N., Yadav S., Rastogi A. (2022). The role of potassium on drought resistance of winter wheat cultivars under cold dryland conditions: Probed by chlorophyll a fluorescence. Plant Physiol. Biochem..

[19] Han M., Wu W., Wu W., Wang Y. (2016). Potassium transporter KUP7 is involved in K^+^ acquisition and translocation in *Arabidopsis* root under K^+^-limited conditions. Mol. Plant.

[20] Maathuis F.J.M. (2009). Physiological functions of mineral macronutrients. Curr. Opin. Plant Biol..

[21] Zhang H., Xiao W., Yu W., Yao L., Li L., Wei J., Li R. (2018). Foxtail millet *SiHAK1* excites extreme high-affinity K^+^ uptake to maintain K^+^ homeostasis under low K^+^ or salt stress. Plant Cell Rep..

[22] Schachtman D.P., Shin R. (2007). Nutrient sensing and signaling: NPKS. Annu. Rev. Plant Biol..

[23] Wang Y., Wu W. (2013). Potassium transport and signaling in higher plants. Annu. Rev. Plant Biol..

[24] Véry A.A., Sentenac H. (2003). Molecular mechanisms and regulation of K^+^ transport in higher plants. Annu. Rev. Plant Biol..

[25] Ahn S.J., Shin R., Schachtman D.P. (2004). Expression of *KT*/*KUP* genes in *Arabidopsis* and the role of root hairs in K^+^ uptake. Plant Physiol..

[26] Grabov A. (2007). Plant KT/KUP/HAK potassium transporters: Single family-multiple functions. Ann. Bot..

[27] Santa-María G.E., Rubio F., Dubcovsky J., Rodríguez-Navarro A. (1997). The *HAK1* gene of barley is a member of a large gene family and encodes a high-affinity potassium transporter. Plant Cell.

[28] Kim E.J., Kwak J.M., Uozumi N., Schroeder J.I. (1998). *AtKUP1*: An *Arabidopsis* gene encoding high-affinity potassium transport activity. Plant Cell.

[29] Feng H., Tang Q., Cai J., Xu B., Xu G., Yu L. (2019). Rice *OsHAK16* functions in potassium uptake and translocation in shoot, maintaining potassium homeostasis and salt tolerance. Planta.

[30] Mäser P., Thomine S., Schroeder J.I., Ward J.M., Hirschi K., Sze H., Talke I.N., Amtmann A., Maathuis F.J., Sanders D. (2001). Phylogenetic relationships within cation transporter families of *Arabidopsis*. Plant Physiol..

[31] Yang Z., Gao Q., Sun C., Li W., Gu S., Xu C. (2009). Molecular evolution and functional divergence of HAK potassium transporter gene family in rice (*Oryza sativa* L.). J. Genet. Genom..

[32] Rehman H.M., Nawaz M.A., Shah Z.H., Daur I., Khatoon S., Yang S.H., Chung G. (2017). In-depth genomic and transcriptomic analysis of five K^+^ transporter gene families in soybean confirm their differential expression for nodulation. Front. Plant Sci..

[33] Gupta M., Qiu X., Wang L., Xie W., Zhang C., Xiong L., Lian X., Zhang Q. (2008). KT/HAK/KUP potassium transporters gene family and their whole-life cycle expression profile in rice (*Oryza sativa*). Mol. Genet. Genom..

[34] Okada T., Nakayama H., Shinmyo A., Yoshida K. (2008). Expression of *OsHAK* genes encoding potassium ion transporters in rice. Plant Biotechnol..

[35] Nieves-Cordones M., Ródenas R., Chavanieu A., Rivero R.M., Martinez V., Gaillard I., Rubio F. (2016). Uneven HAK/KUP/KT protein diversity among angiosperms: Species distribution and perspectives. Front. Plant Sci..

[36] Wang Q., Guan C., Wang P., Lv M., Ma Q., Wu G., Bao A., Zhang J., Wang S. (2015). *AtHKT1;1* and *AtHAK5* mediate low-affinity Na^+^ uptake in *Arabidopsis thaliana* under mild salt stress. Plant Growth Regul..

[37] Osakabe Y., Arinaga N., Umezawa T., Katsura S., Nagamachi K., Tanaka H., Ohiraki H., Yamada K., Seo S.U., Abo M. (2013). Osmotic stress responses and plant growth controlled by potassium transporters in *Arabidopsis*. Plant Cell.

[38] Zhang M., Huang P., Ji Y., Wang S., Wang S., Li Z., Guo Y., Ding Z., Wu W., Wang Y. (2020). KUP9 maintains root meristem activity by regulating K^+^ and auxin homeostasis in response to low K. EMBO Rep..

[39] Zhang H., Yang Z., You X., Heng Y., Wang Y. (2021). The potassium transporter *AtKUP12* enhances tolerance to salt stress through the maintenance of the K^+^/Na^+^ ratio in *Arabidopsis*. Phyton-Int. J. Exp. Bot..

[40] Yang T., Zhang S., Hu Y., Wu F., Hu Q., Chen G., Cai J., Wu T., Moran N., Yu L. (2014). The role of a potassium transporter *OsHAK5* in potassium acquisition and transport from roots to shoots in rice at low potassium supply levels. Plant Physiol..

[41] Chen G., Hu Q., Luo L., Yang T., Zhang S., Hu Y., Yu L., Xu G. (2015). Rice potassium transporter OsHAK1 is essential for maintaining potassium-mediated growth and functions in salt tolerance over low and high potassium concentration ranges. Plant Cell Environ..

[42] Feng X., Liu W., Qiu C., Zeng F., Wang Y., Zhang G., Chen Z., Wu F. (2020). *HvAKT2* and *HvHAK1* confer drought tolerance in barley through enhanced leaf mesophyll H^+^ homoeostasis. Plant Biotechnol. J..

[43] Song Z., Yang Y., Ma R., Xu J., Yu M. (2015). Transcription of potassium transporter genes of KT/HAK/KUP family in peach seedlings and responses to abiotic stresses. Biol. Plant..

[44] Ou W., Mao X., Huang C., Tie W., Yan Y., Ding Z., Wu C., Xia Z., Wang W., Zhou S. (2018). Genome-wide identification and expression analysis of the KUP family under abiotic stress in cassava (*Manihot esculenta* Crantz). Front. Physiol..

[45] Yang X., Zhang J., Wu A., Wei H., Fu X., Tian M., Ma L., Lu J., Wang H., Yu S. (2020). Genome-wide identification and expression pattern analysis of the *HAK*/*KUP*/*KT* gene family of cotton in fiber development and under stresses. Front. Genet..

[46] Zhang H., Gao X., Zhi Y., Li X., Zhang Q., Niu J., Wang J., Zhai H., Zhao N., Li J. (2019). A non-tandem CCCH-type zinc-finger protein, *IbC3H18*, functions as a nuclear transcriptional activator and enhances abiotic stress tolerance in sweet potato. New Phytol..

[47] Wang F., Zhai H., An Y., Si Z., He S., Liu Q. (2016). Overexpression of *IbMIPS1* gene enhances salt tolerance in transgenic sweetpotato. J. Integr. Agric..

[48] Jin R., Jiang W., Yan M., Zhang A., Liu M., Zhao P., Chen X., Tang Z. (2021). Genome-wide characterization and expression analysis of HAK K^+^ transport family in *Ipomoea*. 3 Biotech.

[49] Mittler R., Vanderauwera S., Gollery M., Breusegem F.V. (2004). Reactive oxygen gene network of plants. Trends Plant Sci..

[50] Ayala A., Muñoz M.F., Argüelles S. (2014). Lipid peroxidation: Production, metabolism, and signaling mechanisms of malondialdehyde and 4-hydroxy-2-nonenal. Oxid. Med. Cell. Longev..

[51] Wang Y., Lv J., Chen D., Zhang J., Qi K., Cheng R., Zhang H., Zhang S. (2018). Genome-wide identification, evolution, and expression analysis of the *KT*/*HAK*/*KUP* family in pear. Genome.

[52] Maathuis F.J.M. (2006). The role of monovalent cation transporters in plant responses to salinity. J. Exp. Bot..

[53] Chérel I., Lefoulon C., Boeglin M., Sentenac H. (2014). Molecular mechanisms involved in plant adaptation to low K^+^ availability. J. Exp. Bot..

[54] Gierth M., Mäser P. (2007). Potassium transporters in plants-involvement in K^+^ acquisition, redistribution and homeostasis. FEBS Lett..

[55] Rubio F., Santa-Maria G.E., Rodriguez-Navarro A. (2000). Cloning of *Arabidopsis* and barley cDNAs encoding HAK potassium transporters in root and shoot cells. Physiol. Plant..

[56] Gierth M., Mäser P., Schroeder J.I. (2005). The potassium transporter HAK5 functions in K^+^ deprivation-induced high-affinity K^+^ uptake and *AKT1* K^+^ channel contribution to K^+^ uptake kinetics in *Arabidopsis* roots. Plant Physiol..

[57] Bañuelos M.A., Garciadeblas B., Cubero B., Rodríguez-Navarro A. (2002). Inventory and functional characterization of the HAK potassium transporters of rice. Plant Physiol..

[58] Qi Z., Hampton C.R., Shin R., Barkla B.J., White P.J., Schachtman D.P. (2008). The high affinity K^+^ transporter *AtHAK5* plays a physiological role in planta at very low K^+^ concentrations and provides a caesium uptake pathway in *Arabidopsis*. J. Exp. Bot..

[59] Fu H., Luan S. (1998). *AtKUP1*: A dual-affinity K^+^ transporter from *Arabidopsis*. Plant Cell.

[60] Shabala S. (2017). Signalling by potassium: Another second messenger to add to the list?. J. Exp. Bot..

[61] Hauser F., Horie T. (2010). A conserved primary salt tolerance mechanism mediated by HKT transporters: A mechanism for sodium exclusion and maintenance of high K^+^/Na^+^ ratio in leaves during salinity stress. Plant Cell Environ..

[62] Asada K. (2006). Production and scavenging of reactive oxygen species in chloroplasts and their functions. Plant Physiol..

[63] Shabala S., Cuin T.A. (2008). Potassium transport and plant salt tolerance. Physiol. Plant..

[64] Wu H., Shabala L., Zhou M., Shabala S. (2015). Chloroplast-generated ROS dominate NaCl-induced K^+^ efflux in wheat leaf mesophyll. Plant Signal. Behav..

[65] Zhang F., Wang Y., Yang Y., Wu H., Wang D., Liu J. (2007). Involvement of hydrogen peroxide and nitric oxide in salt resistance in the calluses from *Populus euphratica*. Plant Cell Environ..

[66] Sun J., Li L., Liu M., Wang M., Ding M., Deng S., Lu C., Zhou X., Shen X., Zheng X. (2010). Hydrogen peroxide and nitric oxide mediate K^+^/Na^+^ homeostasis and antioxidant defense in NaCl-stressed callus cells of two contrasting poplars. Plant Cell Tissue Organ Cult..

[67] Zhu H., Zhou Y., Zhai H., He S., Zhao N., Liu Q. (2020). A novel sweetpotato WRKY transcription factor, IbWRKY2, positively regulates drought and salt tolerance in transgenic *Arabidopsis*. Biomolecules.

[68] Clough S.J., Bent A.F. (1998). Floral dip: A simplified method for Agrobacterium-mediated transformation of *Arabidopsis thaliana*. Plant J..

[69] Xu J., Li H., Chen L., Wang Y., Liu L., He L., Wu W. (2006). A protein kinase, interacting with two calcineurin B-like proteins, regulates K^+^ transporter AKT1 in Arabidopsis. Cell.

[70] Du B., Nie N., Sun S., Hu Y., Bai Y., He S., Zhao N., Liu Q., Zhai H. (2021). A novel sweetpotato RING-H2 type E3 ubiquitin ligase gene *IbATL38* enhances salt tolerance in transgenic *Arabidopsis*. Plant Sci..

[71] Zhao L., Liu F., Xu W., Di C., Zhou S., Xue Y., Yu J., Su Z. (2009). Increased expression of *OsSPX1* enhances cold/subfreezing tolerance in tobacco and *Arabidopsis thaliana*. Plant Biotechnol. J..

[72] Fu X., Khan E.U., Hu S., Fan Q., Liu J. (2011). Overexpression of the *betaine aldehyde dehydrogenase* gene from *Atriplex hortensis* enhances salt tolerance in the transgenic trifoliate orange (*Poncirus trifoliata* L. Raf.). Environ. Exp. Bot..

