# Supplementary material for: The Sweet Potato K+ Transporter IbHAK11 Regulates K+ Deficiency and High Salinity Stress Tolerance by Maintaining Positive Ion Homeostasis
Source: Plants (Basel). 2023 Jun 23;12(13):2422. doi: 10.3390/plants12132422 (PMC10346689; doi:10.3390/plants12132422)
Supplement: Supplementary file 1 [file plants-12-02422-s001.zip › plants-2443310-supplementary.pdf]

Table S1. Primers used in this study

Primer name	Primer scequence（5'→3'）
IbActin-F:	AGCAGCATGAAGATTAAGGTTGTAGCAC
IbActin-R:	TGGAAAATTAGAAGCACTTCCTGTGAAC
IbKUP11-qPCR-F:	CCTTTTGAAGATGAATAACAGC
IbKUP11-qPCR-R:	CTGCTTGCTCTAATGACTGTG
IbKUP11-SacI-F:	CGAGCTCATGCTGGTCACCACACTTCTGAT
IbKUP11-XbaI-R:	GCTCTAGATACATAGAAAATCTGTCCAACATTC
IbHAK11-T-F:	GGTCGCGGAGGCTATGGATGC
IbHAK11-T-R:	GCTTCTGCGGGCGATTTGTGT
Atactin-F	GCACCCTGTTCTTCTTACCGA
Atactin-R	AGTAAGGTCACGTCCAGCAAGG
AtCAT-F	GCAACTACCCCGAGTGGAAA
AtCAT-R	TGTTCAGAACCAAGCGACCA
AtGPX8-F	ATGGCGACGAAGGAACCAG
AtGPX8-R	ATCGCCGAAGATTCCCCATTT
AtP5CS-F	ATGATCTTATTTATGTTCTGC
AtP5CS-R	CACTATCTTCCGTCACTAT
AtP5CR-F	AGTTTAGCTTCACAGACCGTTC
AtP5CR-R	GCTCTGTGAGAGCTCGCGGCTTC
AtDHAR-F	ATGGTCCTTTTATCGCCGGG
AtDHAR-R	GCCCATCCAGAGATCACACA
AtAPX-F	CTCTGGGACGATGCCACAAG
AtAPX-R	CTCGACCAAAGGACGGAAAA
AtPOD-F	TCCGGGAGCCACACCATTGG
AtPOD-R	TGGTCGGAATTCAACAG
AtSOD-F	ATGAGAAGTTCTATGAAGAG
AtSOD-R	GTCTTTATGTAATCTGGT

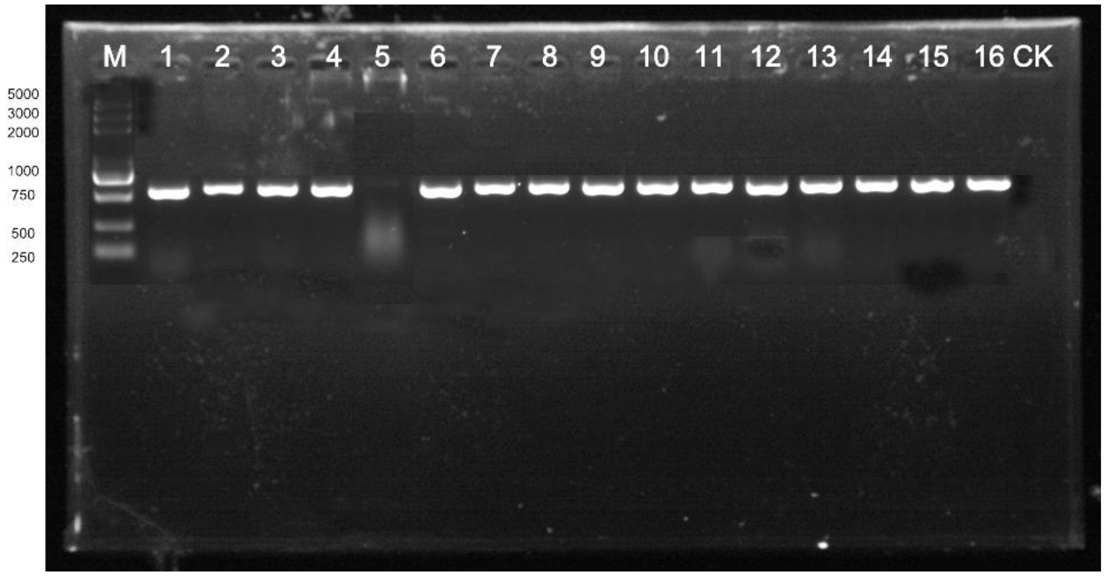

Figure S1. PCR identification of *IbHAK11* transgenic plants. M, DNA marker; CK, negative control; 1-16, candidate transgenic plants.
